# Contrasting Phenological Patterns and Reproductive Strategies in Closely Related Monoecious Fig Tree Species

**DOI:** 10.3390/plants13141889

**Published:** 2024-07-09

**Authors:** Monise T. Cerezini, Ludmila Rattis, Paulo R. Furini, Rodrigo A. S. Pereira

**Affiliations:** 1 Faculdade de Ciências Aplicadas, Universidade Estadual de Campinas, Limeira 13484-350, SP, Brazil; mo_terra@yahoo.com.br; 2 Woodwell Climate Research Center, Tropics Program, Falmouth, MA 02540-1644, USA; ludmilarattis@gmail.com; 3 Instituto de Pesquisa Ambiental da Amazônia, Brasília 70863-520, DF, Brazil; 4 Pós-Graduação em Biologia Comparada, Faculdade de Filosofia, Ciências e Letras de Ribeirão Preto, Universidade de São Paulo, Ribeirão Preto 14040-130, SP, Brazil; prfurini.bio@usp.br; 5 Departamento de Biologia, Faculdade de Filosofia, Ciências e Letras de Ribeirão Preto, Universidade de São Paulo, Ribeirão Preto 14040-130, SP, Brazil

**Keywords:** brood-site pollination, mutualism, plant-insect interaction, reproductive strategy, wasp pollination

## Abstract

Understanding the ecological and evolutionary aspects of mutualistic interactions is essential for predicting species responses to environmental changes. This study aimed to investigate the phenological patterns and reproductive strategies in two closely related fig tree species, *Ficus citrifolia* and *Ficus eximia*. We monitored 99 *F. citrifolia* and 21 *F. eximia* trees weekly from January 2006 to April 2011 in an area close to the southern edge of the tropical region in Brazil. Our results revealed contrasting phenological patterns between the two species, with *F. citrifolia* displaying an annual flowering pattern (1.4 episodes per tree per year) and *F. eximia* a supra-annual pattern (0.5 episodes per tree per year). We also found significant differences in reproductive strategies, with *F. eximia* producing more pistillate flowers and, consequently, more seeds and pollinating wasps per fig than *F. citrifolia*, likely as an adaptation to overcome limitations of low population density by maximizing the gene flow. As the shorter-lived organism, the fig wasp was found to influence critical processes associated with the success and stability of mutualism, such as fig development and ripening. Our findings emphasize the importance of understanding the intricate interactions between mutualistic partners and their adaptive responses to environmental conditions in shaping fig tree populations’ reproductive strategies and genetic structure.

## 1. Introduction

Phenology, the study of the timing of seasonal events and life cycles in plants and animals, plays a critical role in understanding population and community ecology and conservation biology [1,2,3]. Phenological studies are essential for understanding and predicting how intrinsic and extrinsic factors interact to shape the timing and patterns of plant phenological events [4,5]. Temperature can affect bud burst, flowering, and leaf senescence, while precipitation patterns can impact plant growth and development. Photoperiod cues can trigger specific phenological events, especially about the onset of flowering or dormancy [4,5]. Seasonal shifts in temperature, light availability, and moisture levels act as cues for plants to initiate specific phenological phases [5].

Intrinsic factors, including genetic traits and variations within plant populations, can influence phenology. Genetic differences can determine the timing of flowering, leaf-out, and other phenological events [6,7]. The lack of strong climate cues for plants in non-seasonal environments necessitates alternative mechanisms to trigger phenophases. These plants may rely on biotic cues, such as the presence or density of pollinators, herbivores, or neighboring plants, to initiate growth, reproduction, or other life cycle events [8]. For instance, the flowering of some tropical plant species has been linked to the abundance of their primary pollinators, such as bats or birds, and competitive interactions with neighboring plants for light or resources [9,10].

Plant phenology plays a vital role in plant-insect interactions, as it dictates the timing of resource availability for insects and synchronizes their life cycles with those of their host plants [11]. Insect herbivores, pollinators, and parasitoids rely on plant phenophases, such as leafing, flowering, and fruiting, for essential resources such as food, oviposition sites, and shelter [12,13]. In some plant species engaged in obligate and specialized pollinating mutualisms, plant phenology appears to be selected by pollinator availability rather than climate cues [14]. This is particularly true for plants that rely on a single or a small number of specialized pollinator species, as their reproductive success is closely tied to the presence and abundance of these pollinators [15]. Consequently, these plant species may exhibit phenological patterns that are less responsive, or even unresponsive, to the climate. One well-known example of mutualism is the interaction between cultivated/non-cultivated fig trees (*Ficus* spp.) and their specialized pollinating fig wasps (Agaonidae) [16]. Fig trees produce enclosed inflorescences called syconia or fig, which provide a unique and protected environment for fig wasp reproduction. The phenology of monoecious fig trees is often asynchronous at the population level, with individual trees producing syconia at different times, ensuring the continuous availability of receptive inflorescences for the wasps [17,18,19,20,21]. This asynchrony can be maintained even in seasonal environments, supporting that the phenology of these plant species is more closely tied to pollinator presence than to climate factors [22,23]. Similarly, in the yucca-yucca moth mutualism, the phenology of yucca plants (*Yucca* spp.) is synchronized with the emergence of their specialized pollinators, the yucca moths (*Tegeticula* and *Parategeticula*, Prodoxidae, Lepidoptera) [24]. Yucca plants rely exclusively on these moths for pollination, and the moths depend on yucca flowers as a food source and oviposition site for their larvae. The tight coupling of yucca phenology and yucca moth emergence ensures the mutualistic relationship persists.

Differences in phenological patterns across species remain primarily unexplored in monoecious fig trees. Even within the same biome, phylogenetically related *Ficus* species can display contrasting ecological and biological traits [22,25]. For instance, some species are characterized by large trees that produce massive quantities of figs and occur at very low population densities. Conversely, other species consist of smaller trees that produce fewer figs and exhibit higher population densities, often in aggregated distributions [25,26]. However, studies that compare the phenological patterns among fig tree species with such biological and ecological differences remain lacking. Understanding the potential phenological variations among monoecious fig tree species, their implications for pollinator populations, and the stability of the fig-fig wasp mutualism will contribute to a more comprehensive understanding of the ecology and conservation of these keystone species.

Giving the considerations above, our study aimed to investigate the phenology and reproductive traits of two contrasting fig tree species within the section *Americanae* over 64 months. *Ficus citrifolia* P. Miller, a pioneer species, is characterized by moderated-sized trees that exhibit high population densities in disturbed habitats. In contrast, *F. eximia* Schott, classified as a secondary species, consists of large trees at lower population densities. Specifically, we aim to evaluate potential disparities in phenological strategies employed by these two fig tree species. Data regarding the reproductive characteristics of the studied fig trees and their pollinating wasps were utilized to evaluate the plant’s ecological and biological attributes.

## 2. Results

### 2.1. Flowering Patterns

**Figure 1:**
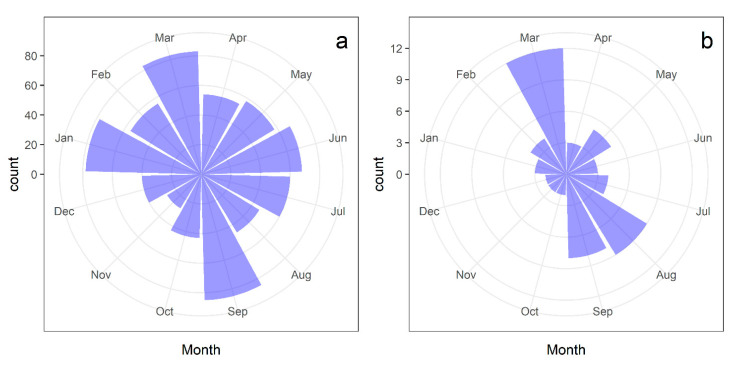
Distribution of fig crop initiation events in *Ficus citrifolia* (**a**) and *Ficus eximia* (**b**) trees over five years. The circular plots illustrate the seasonal patterns of fig crop initiation events, highlighting the distribution of flowering events across different months. The data provides insights into the temporal variability and synchronization of fig crop initiation in the two studied fig tree species.

Both study species exhibited an asynchronous onset of flowering at the population level, with different individuals initiating fig crops throughout the year. A fig crop refers to a distinct episode in which a single fig tree produces a cohort of figs, spanning the period from the initiation of fig development to their maturation. Over five years, we recorded 698 flowering events on the 99 *F. citrifolia* trees. The circular distribution of the number of flowering events significantly differed from the uniform distribution (Watson’s U^2^ test = 0.94, *p* < 0.01, Figure 1a). For *F. eximia*, we observed 57 flowering events on the 21 monitored trees. Fig crop initiations were concentrated in March and August-September, significantly differing from a uniform distribution (Watson’s U^2^ test = 0.21, 0.025 < *p* < 0.05, Figure 1b).

### 2.2. Frequency of Fig Crop Initiation

**Figure 2:**
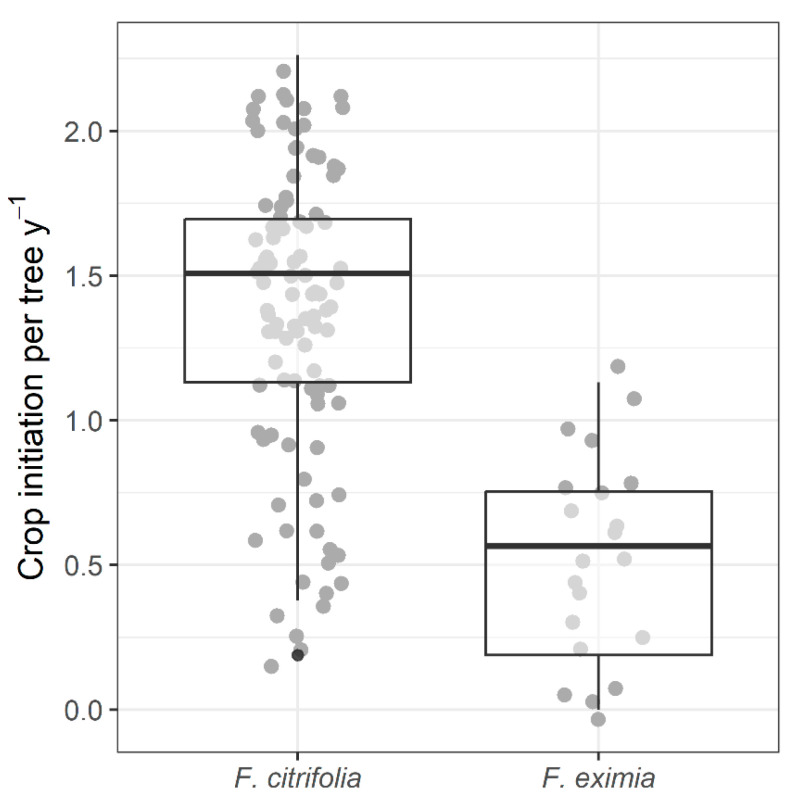
Boxplot of the mean number of crops initiated by each tree per year. Gray dots represent individual trees (values were jittered to improve visualization). Sample sizes: 99 trees for *F. citrifolia* and 21 for *F. eximia*.

**Figure 3:**
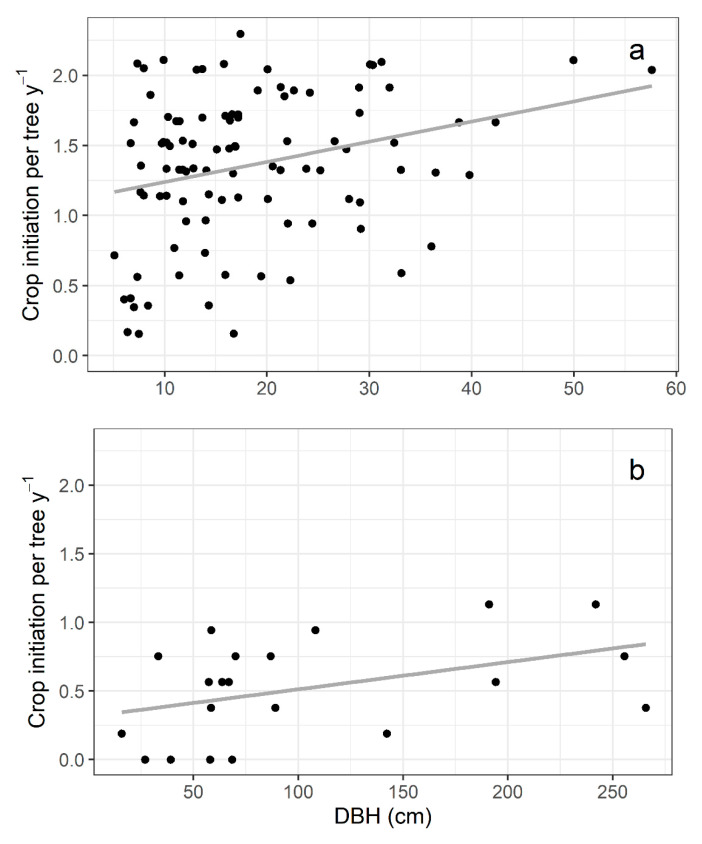
Relationship between number of crops produced per year and trunk diameter at breast height for (**a**) *Ficus citrifolia* and (**b**) *Ficus eximia*. Dots represent individual trees, and the tendency line represents linear regression. Sample sizes: 99 trees for *F. citrifolia* and 21 trees for *F. eximia*.

*Ficus citrifolia* initiated fig crops on average 1.4 ± 0.5 (*n* = 99 trees) times yearly. In comparison, *F. eximia* produced figs 0.5 ± 0.4 (*n* = 21 trees, mean ± SD) times per year (Figure 2). In both species, larger individuals produced figs more times per year. However, there was substantial variance in crop initiation per year, resulting in low R^2^ values (*F. citrifolia*: F_1,97_ = 8.1, *p* = 0.005, R^2^ = 0.08; *F. eximia*: F_1,19_ = 4.2, *p* = 0.055, R^2^ = 0.18; Figure 3). The proportion of trees initiating fig crops per month was not significantly correlated with the studied meteorological variables for either species (Table S1).

### 2.3. Fig Development Time

**Figure 4:**
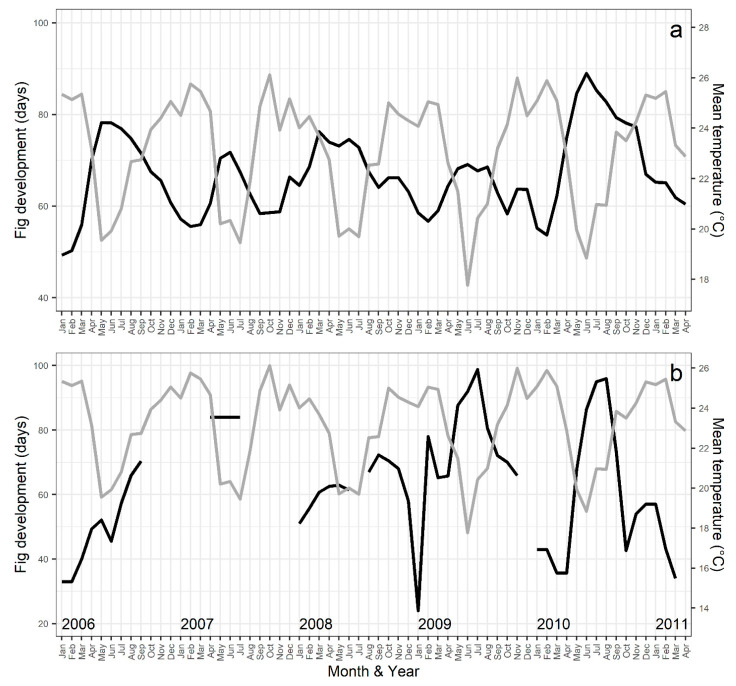
Average time for fig development (black line) and mean monthly temperature (gray line) throughout the study period. (**a**) *F. citrifolia* and (**b**) *F. eximia*.

The time for the fig development, from pre-female to postfloral phases, was not significantly correlated with tree DBH for both species (*F. citrifolia*: F_1,97_ = 0.7, *p* = 0.413; *F. eximia*: F_1,19_ = 2.2, *p* = 0.156). Throughout the 64-month study period, the mean time for fig development varied from 49.2 to 88.9 days for *F. citrifolia* and from 24 to 98.7 days for *F. eximia*, being longer in colder months (Figure 4). However, the mean duration of fig development across the year did not significantly differ between the species (F_1,112_ = 2.4, *p* = 0.125).

In *F. citrifolia*, the time for fig development was significantly negatively correlated with the minimum, median, and maximum temperatures, while in *F. eximia*, it was significantly negatively correlated with the minimum and median temperatures and precipitation (Figures S2 and S3, Table S2).

### 2.4. Reproductive Characteristics and Wasp Community

The analysis revealed no significant difference in fig diameter between the two study species. However, the number of pistillate flowers and seeds was significantly higher in *F. eximia*. The total number of fig wasps did not differ substantially between the species. However, the composition of the wasp community was distinct between the study species. Specifically, *F. eximia* produced a significantly higher number of pollinating wasps, whereas *F. citrifolia* produced a significantly higher number of non-pollinating fig wasps (NPFW). The mesosoma length, representing the pollinator body size, and seed width did not show significant differences between the study species. However, the seeds of *F. citrifolia* were significantly longer than those of *F. eximia* (Table 1).

## 3. Discussion

### 3.1. Phenological Patterns

Fig trees exhibit a unique and evolutionarily successful reproductive biology, resulting from the aggregated dispersion of small pollen and tiny seeds per dispersal unit (i.e., the pollinating agaonid wasp and fig). The strategy of monoecious *Ficus* species to produce large fig crops, resulting in massive production of tiny seeds and pollen-dispersing wasps, seems to allow fig trees to colonize highly transient and infrequent habitats [16]. We observed contrasting phenological patterns between two closely related species, i.e., belonging to the same infrageneric *Ficus* section. Although both species showed asynchronous flowering onset at the population level, at the individual level, *F. citrifolia* had an annual flowering pattern (1.4 episodes). In comparison, *F. eximia* had a supra-annual flowering pattern (0.5 episodes per tree per year on average). In both species, larger individuals were more likely to produce more fig crops yearly.

### 3.2. Reproductive Strategies and Genetic Structure

The differences in flowering frequency between the studied species may reflect contrasting reproductive strategies, impacting their spatial genetic structure. *Ficus eximia* comprises large trees with trunks exceeding 2.5 m in diameter, producing massive numbers of figs during each reproductive episode. As a secondary species, it occurs at a lower relative density than *F. citrifolia*, a pioneer species characterized by medium-sized trees found at high relative densities in disturbed areas or forest edges [25]. The large-tree habit exhibited by some monoecious *Ficus* species, such as *F. eximia*, likely maximizes long-distance seed and pollen dispersal by investing in massive fig production. This strategy may contribute to the weaker spatial genetic structure observed in *F. eximia* across broader spatial scales (approximately 260 km) compared to *F. citrifolia*, which exhibits genetically structured populations on more minor scales (less than 15 km) [27]. The extent of seed dispersal probably has a more substantial impact on the genetic structure of fig tree populations, as seed dispersal (2*n*) contributes twice as much to genetic structuring as pollen dispersal (*n*) when rates of gene flow are similar [28].

### 3.3. Vegetative and Reproductive Growth Rhythms

The phenological patterns observed in the studied species likely reflect differences in the rhythm between vegetative and reproductive growths. Figs are produced in pairs at the leaf axils in both study species, following a phenological pattern. Initially, a shoot develops several internodes, followed by the development of figs at the axils of each leaf. The supra-annual flowering pattern of *F. eximia* suggests that a sequence of shoot growths occurs before fig production or that it produces new shoots less frequently compared to *F. citrifolia* trees. Our findings align with the general patterns seen in tropical plant species. Pioneer species typically undergo selection for rapid growth, early reproductive maturity, and increased reproductive effort, rendering the evolution of supra-annual flowering less likely [29]. The capacity to bloom supra-annually depends on stored reserves, which allow species to produce massive, short-lived displays that effectively recruit pollinators. Understory species, less likely to accumulate such reserves, may not benefit as much from these massive displays, particularly when compared to canopy species that rely on long-distance visual cues to attract pollinators. As a result, supra-annual blooming is expected to be less common in early successional species compared to late successional ones, understory species compared to canopy species, and dioecious species compared to monoecious species [29]. Therefore, the contrasting reproductive strategies of *F. eximia* and *F. citrifolia* can be better understood through their ecological roles, relative abundance, and flowering frequency. This information contributes to a more comprehensive understanding of the evolutionary pressures shaping fig tree populations’ reproductive strategies and genetic structure.

### 3.4. Fig Structure and Mutualism Maintenance

On average, *Ficus eximia* contains 17% more pistillate flowers, resulting in more seeds and pollinating wasps than *F. citrifolia* (see Table 1). The internal infructescence structure and gall packing within the fig accommodate the increased number of seeds and wasps. *Ficus eximia* figs exhibit complete filling of the internal cavity by drupes and galls, a common trait in monoecious species. In contrast, *F. citrifolia* figs do not fill the internal cavity at this stage [30]. The reproductive strategy of *F. eximia*, characterized by massive fig production and efficient seed/gall packing, overcomes the limitations of low population density. This hypothesis highlights the functional role of flower packing in monoecious fig tree species, which can influence pollinator oviposition avoidance because some ovaries are located further from the fig cavity [16,31,32,33].

### 3.5. Coordination of Fig and Wasp Development

**Figure 5:**
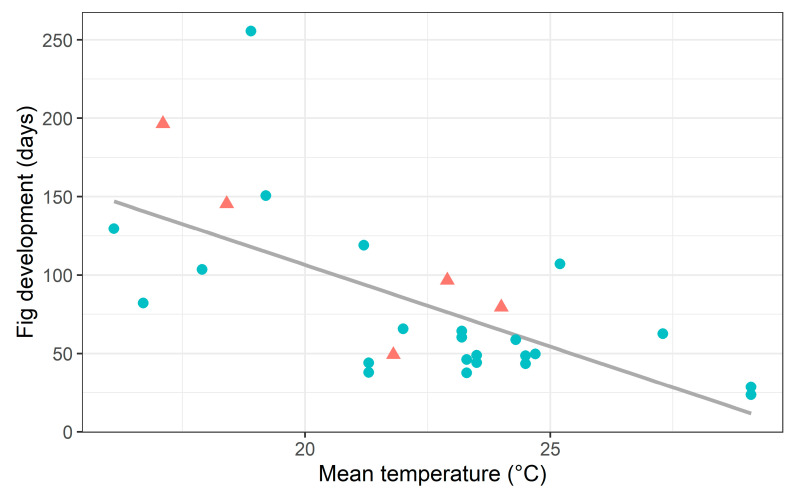
The relationship between fig development period and mean monthly temperature for 12 *Ficus* species, including monoecious (circles) and gynodioecious (triangles) species. For gynodioecious species, the crop duration refers to male trees only. Species names and references are listed in Table S3.

The studied population of *Ficus citrifolia* is associated with the pollinator *Pegoscapus aerumnosus*, while the *Ficus eximia* population is associated with a taxonomically undescribed *Pegoscapus* species. This lack of taxonomic detail is a limitation of the study. However, as we have both morphological and molecular confirmation that the pollinators of the two fig tree species are different, it is still possible to make meaningful biological comparisons. In mutualistic relationships, the larger, long-lived partner often controls the environment, influencing the selective forces acting on the smaller, shorter-lived organism and promoting stability by combining complementary abilities [16]. However, at the individual level, our study highlights that the fig wasp, despite its shorter lifespan, can influence certain aspects of the interaction. For example, the time required for the fig to develop and ripen is likely associated with the larval development and adult activity of the pollinating wasps [34]. Our study and others in the literature have reported a negative correlation between the duration of fig development and environmental temperature [21,23,35,36,37,38,39,40]. In fact, both within and across species, fig development is slower under lower temperatures (Figure 5, Table S3). In gynodioecious species, where wasps develop exclusively in male trees, the total time for fig development in female trees is approximately 7 to 31% longer under similar temperature conditions [39,41,42,43] (Table S4).

Galil and Eisikovitch [34] demonstrated that figs treated with a systemic insecticide, which killed the developing larvae, ripened much later than untreated figs. However, the researchers did not determine whether the ripening process is triggered by the physical injuries inflicted by the wasps when they perforate their galls or chew exit holes, by the cessation of the larvae’s influence, or by combining both factors. Galil and Eisikovitch described a simple and efficient mechanism linking insect activity to fig ripening, which allows for the coordination of wasp and fig development. In the fig-fig wasp mutualism, achieving this coordination might be particularly complex due to the partners belonging to different life realms (i.e., plant and animal) and potentially responding differently to environmental cues.

### 3.6. Environmental Influences on Flowering Patterns

An unresolved issue regarding the distribution of flowering events across months stems from the lack of a biological explanation for the observed pattern in our study. In *F. citrifolia*, the frequency of flowering events was slightly but significantly higher during the drier and colder months (Figure 1). In contrast, another population of *F. citrifolia*, located in a region with an average annual mean temperature 2 °C lower than the study area, exhibited a higher frequency of flowering onset during the rainy season (i.e., November to February) [23]. These contrasting results emphasize the need for further studies to decrypt the underlying mechanisms driving these differences and to enhance our understanding of the adaptive responses of fig trees to varying environmental conditions.

Our study highlights the complexity and intricacies of fig-fig wasp mutualism, revealing contrasting reproductive strategies and phenological patterns between closely related fig tree species. It emphasizes the importance of understanding these species’ adaptive responses to varying environmental conditions and the roles played by both long-lived and short-lived partners in shaping the success and stability of mutualism.

## 4. Materials and Methods

### 4.1. Study Area and Species

Our study occurred between January 2006 and April 2011 at the University of Sao Paulo campus in Ribeirao Preto city (21.166260° S, 47.855183° W). The campus is covered by gardens and lawns, where various spontaneous and ornamental tree species grow. The region’s climate is classified as Aw (Tropical Savanna) according to Köppen’s classification system, with rainy summers and dry winters. The lowest monthly average temperature occurs in July (19.5 °C) and the highest in October (24.8 °C). The area receives an average annual rainfall of 1384 mm, with January being the rainiest month (mean of 256 mm) and July being the driest (mean of 21 mm). Figure S1 presents the climate conditions observed throughout the research period.

*Ficus citrifolia guaranitica*-form is a medium-sized pioneer monoecious species (diameter at breast height (DBH) = 18.3 ± 10.2 cm, mean ± SD, *n* = 99 trees) commonly found as a hemi-epiphyte on other trees or structures, and it frequently thrives in disturbed areas (Figure S4a). In the study area, *F. citrifolia* is pollinated by *Pegoscapus aerumnosus*. *Ficus citrifolia* exhibits substantial morphological variation and has been assigned various names in different regions [44]. The figs grow in pairs at the leaf axils and measure 1.5–2.5 cm in diameter when fully ripe. *Ficus eximia* is a large secondary monoecious tree (DBH = 104.3 ± 78.3 cm, mean ± SD, *n* = 21 trees), and it frequently grows on fallen logs or directly on the ground (Figure S4b). Its population density is considerably lower than that of *F. citrifolia* in forest fragments25 and the study area [45]. In the study area, *F. eximia* is pollinated by an undescribed *Pegoscapus* species. When fully ripe, the figs grow in pairs at the leaf axils and attain a 1.5–2.5 cm diameter. Fernando Farache, a specialist on the Neotropical fig wasp, morphologically identified the pollinating species of both *Ficus* species and confirmed them by comparing mitochondrial COI sequences.

### 4.2. Data Sampling

Over 64 months, we conducted weekly monitoring of reproductive individuals belonging to two species, *F. citrifolia* (*n* = 99) and 21 *F. eximia* (*n* = 21), with a minimum trunk DBH of 5 cm and 15 cm, respectively. Reflecting their respective abundances in the study area, the sample size for *F. citrifolia* was limited to 100 trees, but the final analysis included 99 trees as one was excluded due to being cut down during the study period. For *F. eximia*, due to its lower abundance, we considered all trees that met the inclusion criteria.

During each sampling event, we visually inspected the individuals to determine the presence of figs and assessed their developmental stage based on the classification proposed by Galil and Eisikowitch [46]. These developmental stages encompassed pre-female (young figs before the anthesis of pistillate flowers), female (attractive figs and anthesis of pistillate flowers), interfloral (post-pollination/oviposition, larva and seed development), male (adult wasp offspring emergence and anthesis of staminate flowers), and postfloral phase (departure of pollen-bearing female wasps from the fig, rendering it attractive to frugivorous vertebrates).

Although conducting weekly surveys was necessary for obtaining a more precise measurement of phenophases, as some fig phases last less than a week, we aggregated the data monthly by considering the number of trees initiating fig crops per month. Monthly intervals provided more meaningful biological interpretations and were more suited for statistical analyses due to lower autocorrelation than weekly data. Hence, at the population level, monthly phenological patterns were represented by the proportions of individuals initiating crops and the average duration of fig development in days, from pre-female to post-floral phases, for crops initiated during a particular month. This variable represents the expected fruiting episode duration for an individual initiating a crop within that month. We also estimated the number of flowering onset events per tree per year. We used diameter at breast height (DBH) to approximate tree size. In cases where individuals possessed multiple trunks at breast height, we calculated the sum of the cross-sectional areas of all trunks and back-calculated their diameters.

To investigate the potential correlation between climate and phenological patterns, we obtained meteorological records from the Instituto Agronomico de Campinas (IAC), Ribeirao Preto unit, which is located 5 km away from our study site. The data series included monthly averages of daily mean, minimum, and maximum temperatures (°C) and total rainfall (mm).

To evaluate the reproductive characteristics of the two investigated species, we utilized unpublished data from a related study conducted by our team. This study collected in-nature samples of nearly ripe figs from trees of both study species in semi-deciduous seasonal forest fragments between September 2007 and December 2010. Sampling locations included the cities of Ribeirao Preto (21.166° S, 47.800° W), Galia (22.400° S, 49.700° W), Teodoro Sampaio (22.433° S, 52.300° W), and nearby areas. We collected 10 and 17 fig crops of *F. eximia* and *F. citrifolia* for each sampling location, respectively. For each fig crop, 20 figs were placed individually in 50 mL plastic flasks until the wasps emerged. We gathered all emerged wasps from each fig, and any un-emerged wasps were extracted from the galls. This allowed us to count the number of wasps of each species in each Figure Additionally, we quantified the number of seeds and pistillate flowers, which were estimated by summing unused flowers (i.e., flowers that were neither pollinated nor oviposited), seeds, empty galls (i.e., bladders—galls in which the larva died during development), and developed galls (i.e., the total number of wasps, as one wasp emerges from each gall).

We estimated the body size of pollinating fig wasps and seed size for each *Ficus* species, as these may be linked to pollen/seed dispersion. We randomly selected 30 wasp individuals and 30 seeds from three different fig crops of each species (10 wasps/seeds per species). Measurements were performed using a Leica MZ16 stereoscope equipped with a digital camera and a computer workstation running Leica Application Suite (LAS) V3.6 imaging software.

We conducted a literature search on the phenology of figs and selected only studies that presented fig development duration and corresponding mean daily temperature. This resulted in eight studies encompassing data on 12 *Ficus* species.

Voucher specimens from the study plants were deposited at the Herbarium of the Faculty of Philosophy, Sciences, and Letters of Ribeirao Preto (SPFR), University of Sao Paulo—USP (*F. citrifolia*: col. S.P. Teixeira et al. 79; *F. eximia*: col. R.A.S. Pereira et al. 138). R.A.S Pereira identified both voucher specimens.

### 4.3. Data Analyses

For both studied species, we performed the Watson’s U^2^ Test to assess whether the distribution of the number of flowering events throughout the year differed from the uniform distribution. For this analysis, we used the function ‘watson.test’ from the ‘circular’ package in the R software version 4.3.0 [47].

We used linear models to test whether the mean number of flowering events per tree per year and the time for fig development correlated with *Ficus* species or tree DBH. For the latter, we performed separate analyses for each fig tree species. We used generalized linear models (GLM) with quasibinomial error structure to test whether the proportion of trees initiating crops per month correlated with meteorological variables since preliminary analysis detected that model residuals were overdispersed. We performed separate analyses for each explanatory variable. We tested for autocorrelation in the model residuals using autocorrelation function (ACF) plots. No significant autocorrelation was detected for all GLMs. All these analyses were performed using R software.

We used generalized least squares (GLS) for both study species to test whether the time for fig development was related to the meteorological variables. We initially fit simple regressions to check the residuals for autocorrelation using ACF plots. As we detected a significant autocorrelation structure of order 1 for all models, we adjusted the GLS models by incorporating the AR1 autoregressive model. GLS analyses were conducted using the ‘gls’ function from the ‘nlme’ package in the R software.

The reproductive characteristics, including the number of pistillate flowers, total wasps, pollinating wasps, non-pollinating wasps, and seeds per fig/crop, as well as mesosoma length, seed length, and width, were compared between the fig tree species using linear models in the R software.

## Figures and Tables

**Table 1:** Reproductive characteristics per fig of *Ficus citrifolia* and *F. eximia*. N represents the number of fig crops used to estimate means and standard deviations (SD), except for mesosoma and seed sizes, where N represents the number of measured individuals. For each fig crop, variable values were derived from the average of 15–20 sampled figs. PolFW = pollinating fig wasps; NPFW = non-pollinating fig wasps; Prob. = ANOVA probability for variable comparisons between fig tree species.

Variables	*F. citrifolia*		*F. eximia*		Prob.
	N	Mean ± SD	N	Mean ± SD	
No of pistillate flowers	51	276.0 ± 102.2	30	331.0 ± 141.6	0.047
No of wasps (total)	51	136.3 ± 51.2	30	159.2 ± 72.8	0.101
No of PolFW	51	71.4 ± 67.0	30	114.2 ± 86.0	0.014
No of NPFW	51	64.9 ± 39.5	30	45.0 ± 29.3	0.019
No of Seeds	51	112.0 ± 63.7	30	140.8 ± 90.4	0.022
Fig diameter (cm)	51	1.41 ± 0.19	30	1.40 ± 0.15	0.886
Mesosoma length (mm)	30	0.66 ± 0.05	30	0.67 ± 0.05	0.699
Seed length (mm)	30	1.59 ± 0.30	30	1.43 ± 0.13	0.006
Seed width (mm)	30	1.13 ± 0.12	30	1.13 ± 0.11	0.883

## Data Availability

The data are available at https://repositorio.uspdigital.usp.br (accessed on 8 July 2024). To access the data, search using any author name (ex. ‘Cerezini’).

## Supplementary Materials

The following supporting information can be downloaded at: https://www.mdpi.com/article/10.3390/plants13141889/s1, Figure S1. Climate conditions observed throughout the research period. Bars represent rainfall and the grey line represents the mean monthly temperature. The gray area represents the limits between min and max temperatures; Figure S2. Relationship between the time for fig development of *Ficus citrifolia* and environmental variables; Figure S3. Relationship between the time for fig development of *Ficus eximia* and environmental variables; Figure S4. Trees of *Ficus citrifolia* (a) and *F. eximia* (b), representing the species habitus and the fig branch (bottom-right corner); Table S1. Results of generalized linear models (quasibinomial family) of the proportion of trees initiating crops × month^−1^ as a function of environmental variables; Table S2. Results of generalized least squares (correlation structure: corARMA (*p* = 1)) of the crop duration in days as a function of environmental variables; Table S3. Data on duration of fig development under different environmental temperatures for 12 *Ficus* species; Table S4. Duration of fig development in female and male trees for four *Ficus* species. References [48,49,50] are cited in the supplementary materials.
