# Supplementary material for: Contrasting Phenological Patterns and Reproductive Strategies in Closely Related Monoecious Fig Tree Species
Source: Plants (Basel). 2024 Jul 9;13(14):1889. doi: 10.3390/plants13141889 (PMC11280309; doi:10.3390/plants13141889)
Supplement: Supplementary file 1 [file plants-13-01889-s001.zip › plants-3090276-supplementary.pdf]

Supplementary material

Contrasting phenological patterns and reproductive strategies in closely related monoecious fig tree species

Monise T. Cerezini[1], Ludmila Rattis[2,3], Paulo R. Furini[4] and Rodrigo A. S. Pereira[5*]

1 Faculdade de Ciências Aplicadas, Universidade Estadual de Campinas, Limeira, SP, 13484-350, Brazil; mo_terra@yahoo.com.br
2 Woodwell Climate Research Center, Tropics Program, Falmouth, MA, 02540-1644, USA; ludmilarattis@gmail.com
3 Instituto de Pesquisa Ambiental da Amazônia, Brasília, DF, 70863-520, Brazil
4 Pós-Graduação em Biologia Comparada, Faculdade de Filosofia, Ciências e Letras de Ribeirão Preto, Universidade de São Paulo, Ribeirão Preto, SP, 14040-130, Brazil; prfurini.bio@usp.br
5 Departamento de Biologia, Faculdade de Filosofia, Ciências e Letras de Ribeirão Preto, Universidade de São Paulo, Ribeirão Preto, SP, 14040-130, Brazil
* Correspondence: raspereira@usp.br

Ethical Issues

The study on plant and insect samples was conducted under the licenses for plant material/specimen collection (# 10930-1) and for zoological material/specimen collection (# 10657-1), obtained by R.A.S Pereira from the Instituto Brasileiro do Meio Ambiente e dos Recursos Naturais Renováveis – IBAMA. All research methodologies adhered to the regulations of the Brazilian legislation, the IUCN Policy Statement on Research Involving Species at Risk of Extinction, and the Convention on the Trade in Endangered Species of Wild Fauna and Flora.

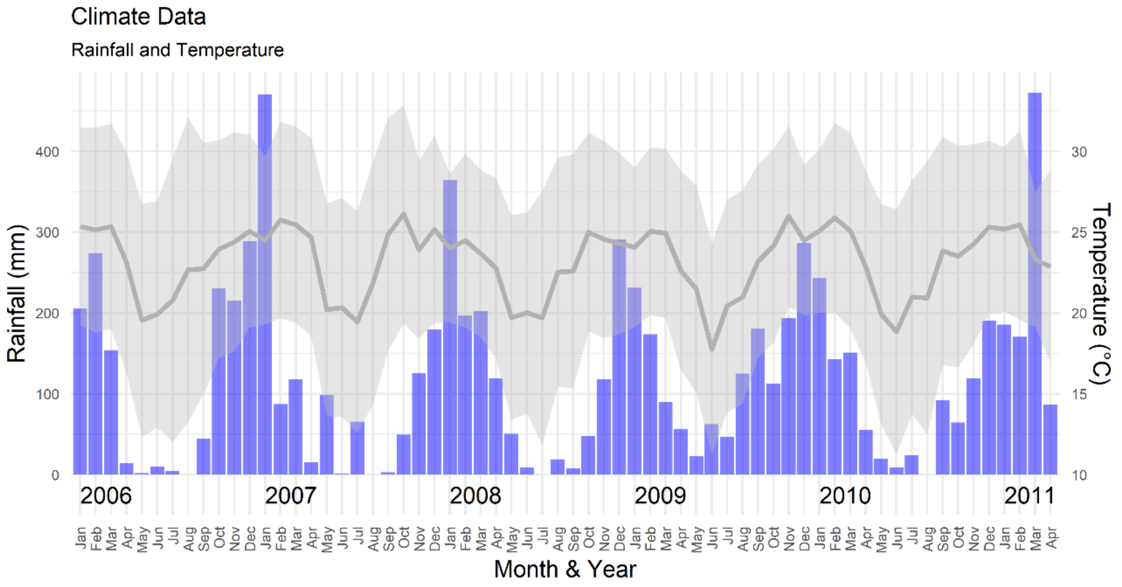

Figure S1. The climate conditions observed throughout the research period. Bars represent rainfall, and the grey line represents the mean monthly temperature. The gray area represents the limits between minimum and maximum temperatures.

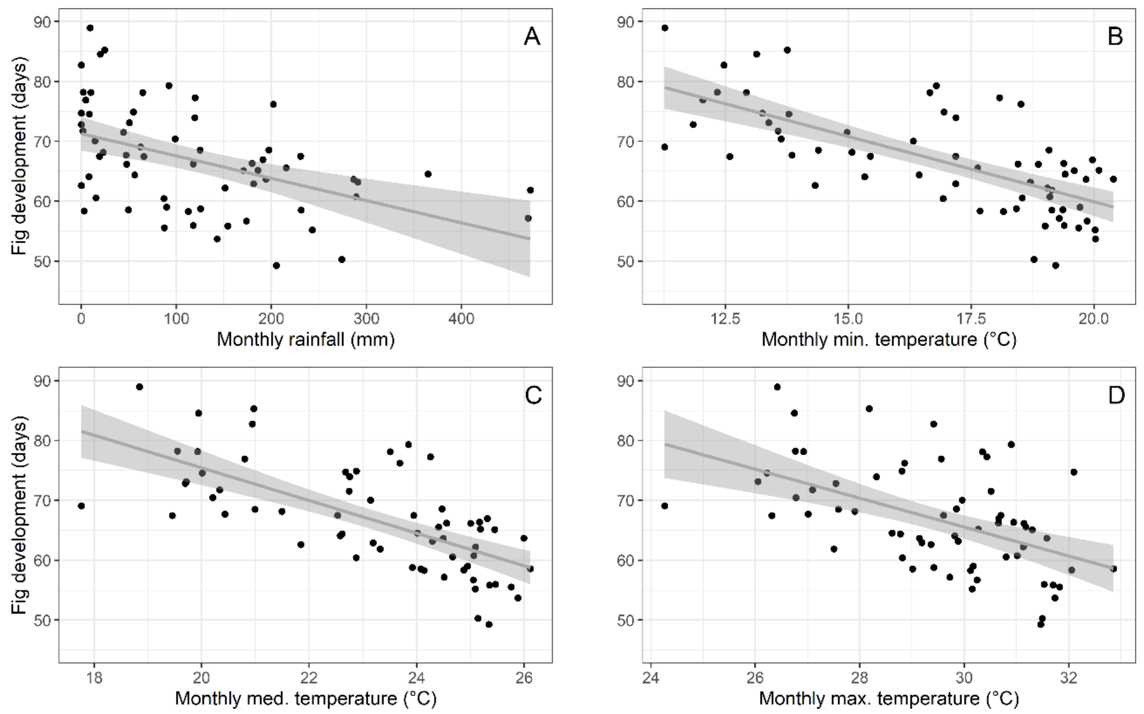

Figure S2. Relationship between the time for fig development of *Ficus citrifolia* and environmental variables.

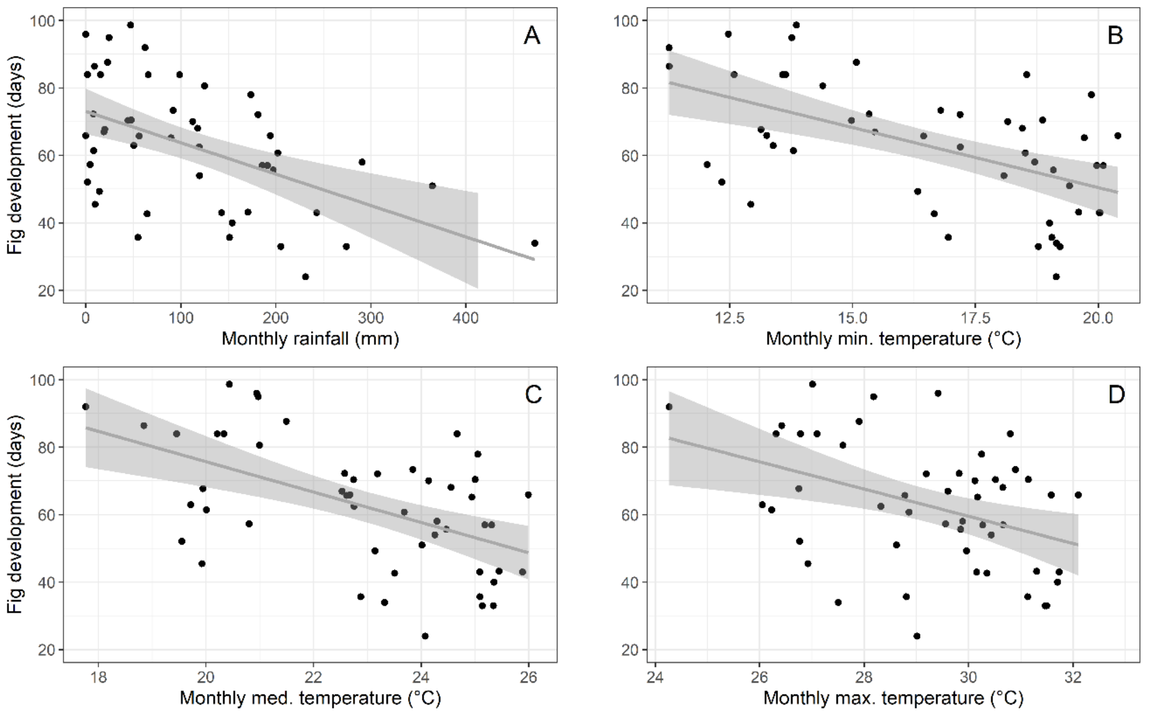

Figure S3. Relationship between the time for fig development of *Ficus eximia* and environmental variables.

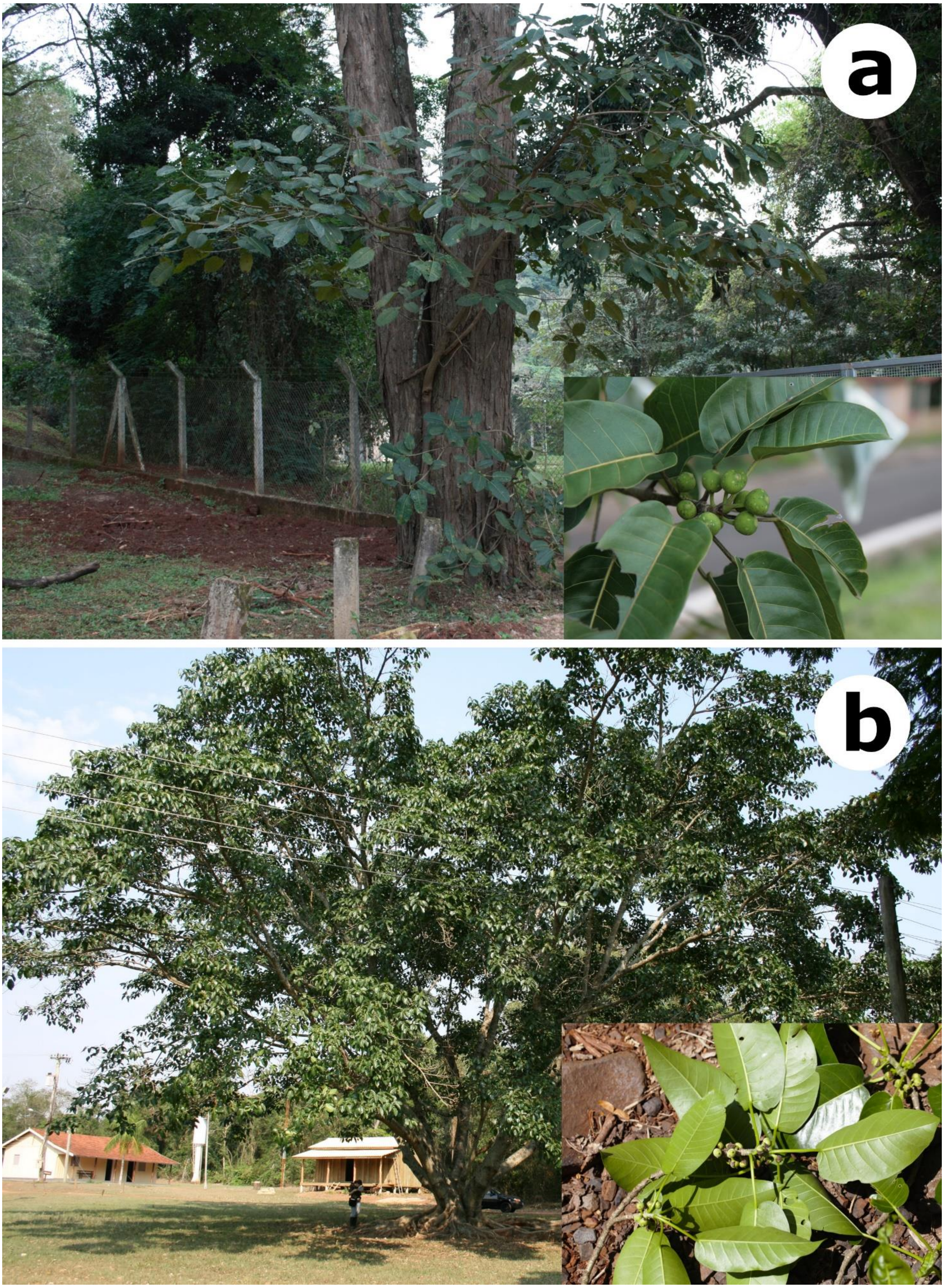

Figure S4. Trees of *Ficus citrifolia* (**a**) and *F. eximia* (**b**), representing the species habitus and the fig branch (bottom-right corner).

Table S1. Results of generalized linear models (quasibinomial family) of the proportion of trees initiating crops × month^{-1} as a function of environmental variables.

**Model**	**Slope**	***t* value**	***P* value**
Ficus citrifolia			
Rainfall	-0.0002	-0.313	0.756
Min. temperature	-0.025	-0.998	0.322
Max. temperature	-0.028	-0.733	0.466
Med. temperature	-0.032	-0.963	0.340
Ficus eximia			
Rainfall	-0.0004	-0.237	0.813
Min. temperature	-0.029	-0.421	0.675
Max. temperature	-0.107	-1.079	0.285
Med. temperature	-0.064	-0.734	0.466

Table S2. Results of generalized least squares (correlation structure: corARMA(p=1)) of the crop duration in days as a function of environmental variables.

**Model**	**Slope**	**t value**	**P value**
Ficus citrifolia			
Rainfall	-0.010	-1.692	0.096
Min. temperature	-1.454	-5.325	$< 10^{-15}$
Med. temperature	-1.620	-4.949	$< 10^{-15}$
Max. temperature	-1.184	-3.423	0.001
Ficus eximia			
Rainfall	-0.069	-3.200	0.002
Min. temperature	-2.495	-2.781	0.008
Med. temperature	-2.353	-2.023	0.049
Max. temperature	-0.659	-0.503	0.617

Table S3. Data on the duration of fig development under different environmental temperatures for 12 *Ficus* species.

Species	Reproductive system	Latitude	Mean temp (C)	Duration (days)	Reference
F. adhatodifolia	monoecious	-23.4500	21.3	44.0	[1]
F. altissima	monoecious	21.9167	27.3	62.6	[2]
F. altissima	monoecious	21.9167	19.2	150.7	[2]
F. altissima	monoecious	25.8333	25.2	107.0	[2]
F. altissima	monoecious	25.8333	18.9	255.5	[2]
F. aurea	monoecious	25.7170	29.1	28.6	[3]
F. aurea	monoecious	25.7170	23.3	46.1	[3]
F. caulocarpa	monoecious	21.9600	23.5	48.8	[4]
F. citrifolia	monoecious	-23.3200	21.2	118.9	[5]
F. citrifolia	monoecious	-21.1663	23.2	64.2	This study
F. citrifolia	monoecious	25.7170	29.1	23.6	[3]
F. citrifolia	monoecious	25.7170	23.3	37.7	[3]
F. eximia	monoecious	-23.4500	21.3	38.0	[1]
F. eximia	monoecious	-21.1663	23.2	60.3	This study
F. racemosa	monoecious	-19.2583	24.5	43.5	[6]
F. racemosa	monoecious	21.6833	22.0	65.7	[7]
F. racemosa	monoecious	21.9167	24.7	49.7	[2]
F. racemosa	monoecious	21.9167	17.9	103.5	[2]
F. racemosa	monoecious	25.8333	24.3	58.7	[2]
F. racemosa	monoecious	25.8333	16.1	129.5	[2]
F. rubiginosa	monoecious	-19.2583	24.5	48.5	[6]
F. subpisocarpa	monoecious	21.9600	23.5	44.1	[4]
F. virens	monoecious	31.4833	16.7	82.1	[8]
F. hirta	gynodioecious	23.1834	21.8	49.3	[9]
F. semicordata	gynodioecious	21.9167	24.0	79.5	[2]
F. semicordata	gynodioecious	21.9167	18.4	145.5	[2]
F. semicordata	gynodioecious	25.8333	22.9	96.7	[2]
F. semicordata	gynodioecious	25.8333	17.1	196.5	[2]

Table S4. Duration of fig development in female and male trees for four *Ficus* species.

Species	Sex	Latitude	Mean temp (C)	Duration (days)	Difference (f-m) %	Reference
F. fistulosa	female	1.2833	NA	70	31.4	[10]
F. fistulosa	male	1.2833	NA	48		[10]
F. grossularioides	female	1.2833	NA	70	7.1	[11]
F. grossularioides	male	1.2833	NA	65		[11]
F. hirta	female	23.1834	21.8	61.0	19.2	[9]
F. hirta	male	23.1834	21.8	49.3		[9]
F. semicordata	female	21.9167	23.9	104.2	23.7	[2]
F. semicordata	male	21.9167	24.0	79.5		[2]
F. semicordata	female	25.8333	20.9	127.0	23.9	[2]
F. semicordata	male	25.8333	22.9	96.7		[2]

References

1. Bianchini, E.; Emmerick, J.M.; Messetti, A.V.L.; Pimenta, J.A. Phenology of Two Ficus Species in Seasonal Semi-Deciduous Forest in Southern Brazil. *Braz. J. Biol.* **2015**, *75*, 206–214, doi:10.1590/1519-6984.10614.
2. Chen, H.; Zhang, Y.; Peng, Y.; Corlett, R.T. Latitudinal Effects on Phenology near the Northern Limit of Figs in China. *Sci. Rep.* **2018**, *8*, doi:10.1038/s41598-018-22548-7.
3. Bronstein, J.L.; Patel, A. Temperature-Sensitive Development: Consequences for Local Persistence of Two Subtropical Fig Wasp Species. *Am. Midl. Nat.* **1992**, *128*, 397–403, doi:10.2307/2426473.
4. Huang, Y.-T.; Lee, Y.-F.; Kuo, Y.-M.; Chang, S.-Y.; Wu, C.-L. Fruiting Phenology and Nutrient Content Variation among Sympatric Figs and the Ecological Correlates. *Bot. Stud.* **2019**, *60*, doi:10.1186/s40529-019-0275-9.
5. Pereira, R.A.S.; Rodrigues, E.; Menezes Jr, A.O. Phenological Patterns of Ficus Citrifolia (Moraceae) in a Seasonal Humid-Subtropical Region in Southern Brazil. *Plant Ecol.* **2007**, *188*, 265–275, doi:10.1007/s11258-006-9161-0.
6. Jia, X.C.; Yao, J.Y.; Chen, Y.Z.; Cook, J.M.; Crozier, R.H. The Phenology and Potential for Self-Pollination of Two Australian Monoecious Fig Species. *Symbiosis* **2008**, *45*, 91–96.
7. Zhang, G.M.; Song, Q.S.; Yang, D.R. Phenology of Ficus Racemosa in Xishuangbanna, Southwest China. *Biotropica* **2006**, *38*, 334–341, doi:10.1111/j.1744-7429.2006.00150.x.
8. Zhang, L.-S.; Compton, S.G.; Xiao, H.; Lu, Q.; Chen, Y. Living on the Edge: Fig Tree Phenology at the Northern Range Limit of Monoecious Ficus in China. *Acta Oecologica* **2014**, *57*, 135–141, doi:10.1016/j.actao.2013.09.002.
9. Yu, H.; Zhao, N.X.; Chen, Y.Z.; Deng, Y.; Yao, J.Y.; Ye, H.G. Phenology and Reproductive Strategy of a Common Fig in Guangzhou. *Bot. Stud.* **2006**, *47*, 435–441.
10. Corlett, R.T. The Phenology of Ficus Fistulosa in Singapore. *Biotropica* **1987**, *19*, 122–124.
11. Corlett, R.T. Sexual Dimorphism in the Reproductive Phenology of Ficus Grossularioides Burm. f. in Singapore. *Malay. Nat. J.* **1993**, *46*, 149–155.
